# Antibiotic Prescriptions and Prophylaxis in Italian Children. Is It Time to Change? Data from the ARPEC Project

**DOI:** 10.1371/journal.pone.0154662

**Published:** 2016-05-16

**Authors:** Maia De Luca, Daniele Donà, Carlotta Montagnani, Andrea Lo Vecchio, Marta Romanengo, Claudia Tagliabue, Chiara Centenari, Patrizia D’Argenio, Rebecca Lundin, Carlo Giaquinto, Luisa Galli, Alfredo Guarino, Susanna Esposito, Mike Sharland, Ann Versporten, Herman Goossens, Giangiacomo Nicolini

**Affiliations:** 1 Immunology and Infectious Diseases Unit, University Hospital Pediatric Department, Bambino Gesù Children's Hospital, Rome, Italy; 2 Division of Pediatric Infectious Diseases, Department for Woman and Child Health, University of Padua, Padua, Italy; 3 Paediatric Infectious Diseases Unit, Department of Paediatric Medicine, Anna Meyer Children's University Hospital, Florence, Italy; 4 Department of Translational Medical Sciences—Section of Pediatrics, University of Naples Federico II, Naples, Italy; 5 Acute Care and Emergency Department, G. Gaslini Children's Hospital, Genoa, Italy; 6 Pediatric Highly Intensive Care Unit, Department of Pathophysiology and Transplantation, Università degli Studi di Milano, Fondazione IRCCS Ca' Granda Ospedale Maggiore Policlinico, Milano, Italy; 7 Paediatric Unit, Versilia Hospital, Lido di Camaiore, Italy; 8 Infection and Immunity, Division of Clinical Sciences, St. Georges University of London, London, United Kingdom; 9 Laboratory of Medical Microbiology, Vaccine & Infectious Disease Institute, University of Antwerp, Antwerp, Belgium; 10 Pediatric Unit, San Martino Hospital, Belluno, Italy; Azienda ospedaliero-universitaria di Perugia, ITALY

## Abstract

**Background:**

Antimicrobials are the most commonly prescribed drugs. Many studies have evaluated antibiotic prescriptions in the paediatric outpatient but few studies describing the real antibiotic consumption in Italian children’s hospitals have been published. Point-prevalence survey (PPS) has been shown to be a simple, feasible and reliable standardized method for antimicrobials surveillance in children and neonates admitted to the hospital. In this paper, we presented data from a PPS on antimicrobial prescriptions carried out in 7 large Italian paediatric institutions.

**Methods:**

A 1-day PPS on antibiotic use in hospitalized neonates and children was performed in Italy between October and December 2012 as part of the Antibiotic Resistance and Prescribing in European Children project (ARPEC). Seven institutions in seven Italian cities were involved. The survey included all admitted patients less than 18 years of age present in the ward at 8:00 am on the day of the survey, who had at least one on-going antibiotic prescription. For all patients data about age, weight, underlying disease, antimicrobial agent, dose and indication for treatment were collected.

**Results:**

The PPS was performed in 61 wards within 7 Italian institutions. A total of 899 patients were eligible and 349 (38.9%) had an on-going prescription for one or more antibiotics, with variable rates among the hospitals (25.7% - 53.8%). We describe antibiotic prescriptions separately in neonates (<30 days old) and children (> = 30 days to <18 years old). In the neonatal cohort, 62.8% received antibiotics for prophylaxis and only 37.2% on those on antibiotics were treated for infection. Penicillins and aminoglycosides were the most prescribed antibiotic classes. In the paediatric cohort, 64.4% of patients were receiving antibiotics for treatment of infections and 35.5% for prophylaxis. Third generation cephalosporins and penicillin plus inhibitors were the top two antibiotic classes. The main reason for prescribing antibiotic therapy in children was lower respiratory tract infections (LRTI), followed by febrile neutropenia/fever in oncologic patients, while, in neonates, sepsis was the most common indication for treatment. Focusing on prescriptions for LRTI, 43.3% of patients were treated with 3rd generation cephalosporins, followed by macrolides (26.9%), quinolones (16.4%) and carbapenems (14.9%) and 50.1% of LRTI cases were receiving more than one antibiotic. For neutropenic fever/fever in oncologic patients, the preferred antibiotics were penicillins with inhibitors (47.8%), followed by carbapenems (34.8%), aminoglycosides (26.1%) and glycopeptides (26.1%). Overall, the 60.9% of patients were treated with a combination therapy.

**Conclusions:**

Our study provides insight on the Italian situation in terms of antibiotic prescriptions in hospitalized neonates and children. An over-use of third generation cephalosporins both for prophylaxis and treatment was the most worrisome finding. A misuse and abuse of carbapenems and quinolones was also noted. Antibiotic stewardship programs should immediately identify feasible targets to monitor and modify the prescription patterns in children’s hospital, also considering the continuous and alarming emergence of MDR bacteria.

## Background

Antimicrobials are the most commonly prescribed drugs in the community and hospital setting, especially among paediatric patients [1]. However, antibiotics are often unnecessarily used both in the community, where too many children receive broad-spectrum antibiotics for viral infections, and in the hospital, where long courses of broad-spectrum antibiotics are frequently prescribed [2]. Recent studies have found that up to 50% of antimicrobial prescriptions are inappropriate [3,4].

The emergence of multi-drug resistant (MDR) pathogens and their rapid global spread, strictly associated with an inappropriate use of antimicrobials, are important global public health threats with a substantial impact on patient outcomes such as hospital length of stay and mortality, as well as on healthcare costs [5–8]. The European Antimicrobial Resistance Surveillance Network (EARS-Net) system has reported a dangerous rise in MDR bacteria in the last years showing that some countries such as Italy are strongly contributing to this worrying increase [9].

Many studies have evaluated antibiotic prescriptions in the paediatric outpatient population highlighting the problem that Italian prescribing habits that differ from those of other European countries. An Italian child is more likely to be exposed to antibiotics than children are in North Europe [10] and, in particular, the prevalence of antibiotic prescriptions in childhood have been reported to be 4 times higher than in the UK and 6 times higher than in the Netherlands [11,12]. Moreover, Italy reported the highest prescription rate (1.3 per infants per year) in a study comparing antibiotic use in the first year of life in five European countries [13]. In fact, data from the Gagliotti et al study in 2006 show that the 55% of Italian infants in the community have already received at least one course of antibiotics at 1 year of age and 84% at 2 years of age [14].

Although a positive correlation between outpatient and inpatient antibiotic use has been noted [15], few studies describing the real antibiotic consumption in Italian children’s hospitals have been published. A single centre study was carried out in Rome in 2008 [16] confirming the abuse of antibiotics observed in the outpatient population. A more recent paper evaluating the trend of antibiotic use in all the paediatric wards of Emilia-Romagna Region over an 8-year-period [17] indicated a slight increase of antibiotic consumption over time, an inadequate tendency to prefer penicillin plus inhibitors to plain penicillins, an over-use of third generation cephalosporins and a worrisome increase in linezolid prescriptions.

In this paper, we present the results of a point-prevalence survey (PPS) on antibiotic prescriptions carried out in seven large Italian paediatric institutions in 2012. The aims of our study were: i) to describe prevalence rates of antibiotic prescriptions for prophylaxis and treatment of infections for neonatal (<30 days) and paediatric (age ≥30 days) patients in seven Italian centers; ii) to evaluate antibiotic prescriptions, indications, number and type of antibiotic agents and administration route in the same age sub-groups both for prophylaxis and treatment of infections; iii) to describe over-all consumption and off-label use of particular classes of antibiotics, such as carbapenems and quinolones, in our cohort; and iiii) to identify targets for improving the quality of antimicrobial prescribing in these centers.

## Methods

This research has been conducted according to the principles expressed in the Declaration of Helsinki. Ethical approval has been obtained for the coordinating centre. No consent was given, because data were collected by reviewing medical charts and were analyzed anonymously. Every patient record was given a unique non-identifiable survey number, which was automatically generated by a computer program specifically designed for anonymous data entry.

A 1-day PPS on antibiotic use in hospitalized children was performed in Italy between October and December 2012 as part of the Antibiotic Resistance and Prescribing in European Children project (ARPEC). Seven paediatric or mixed adult-paediatric hospitals in seven Italian cities were involved (Genoa, Milan, Padua, Florence, Viareggio, Rome and Naples). The survey included all admitted patients less than 18 years of age present in the ward at 8:00 am on the day of the survey who had at least one on-going antibiotic prescription. The wards of admission were: medical (general neonatal and maternal wards, and general paediatric wards), special medical (cardiology, nephrology, onco-hematology, neuromuscolary, neurology, bronchopneumology, infectious diseases unit), neonatal and paediatric intensive care (NICUs and PICUs), surgical (neonatal surgery, paediatric surgery, orthopedics, neurosurgery). For feasibility reasons, one hospital provided data from randomly selected wards, maintaining the patient distribution among medical, special medical, surgical and intensive care units, in agreement with the coordinating centre. Full details of the ARPEC methodology are described elsewhere [18].

## Results

The PPS was performed in 61 wards within seven Italian institutions. Characteristics of the centres involved are shown in Table 1. A total of 899 patients was present in the hospitals at 8:00 am on the day of the survey and 349 (38.9%) of these had an on-going prescription for one or more antibiotics. However, this rate was variable among the hospitals ranging from 25.7% to 53.8% (Table 1). Combination therapies were variably used among the institutions (21.7–60.3%) with a ratio between number of prescribed antibiotics and treated patients ranging from 1.25 to 1.76 (Table 1).

**Table 1:** Characteristics of the 7 Italian institutions involved in the ARPEC project.

City	Hospital Characteristics	Treated patients	Total patients	Rates of treatment	Beds	Bed occupancy	N° of prescribed antibiotics	N° of prescribed antibiotics/treated patients	Combination therapies	Combination therapies/treated patients
Rome	Teaching hospital, tertiary hospital	63	117	53.8%	136	86.0%	111	1.76	38	60.3%
Padua	Teaching hospital, tertiary hospital	70	185	37.8%	213	86.9%	124	1.77	36	51.4%
Florence	Teaching hospital, tertiary hospital	59	144	41.0%	169	85.2%	98	1.66	26	44.1%
Milan	Teaching hospital, specialized hospital	38	100	38.0%	128	78.1%	55	1.45	14	36.8%
Genoa	Teaching hospital, tertiary hospital	83	217	38.2%	314	69.1%	104	1.25	18	21.7%
Naples	Teaching hospital, specialized hospital	28	109	25.7%	122	89.3%	42	1.50	11	39.3%
Viareggio	Secondary hospital	8	27	29.6%	45	60.0%	9	1.13	1	12.5%
**TOT**	-	349	899	38.8%	1127	79.8%	543	1.56	144	41.3%

**Fig 1:**
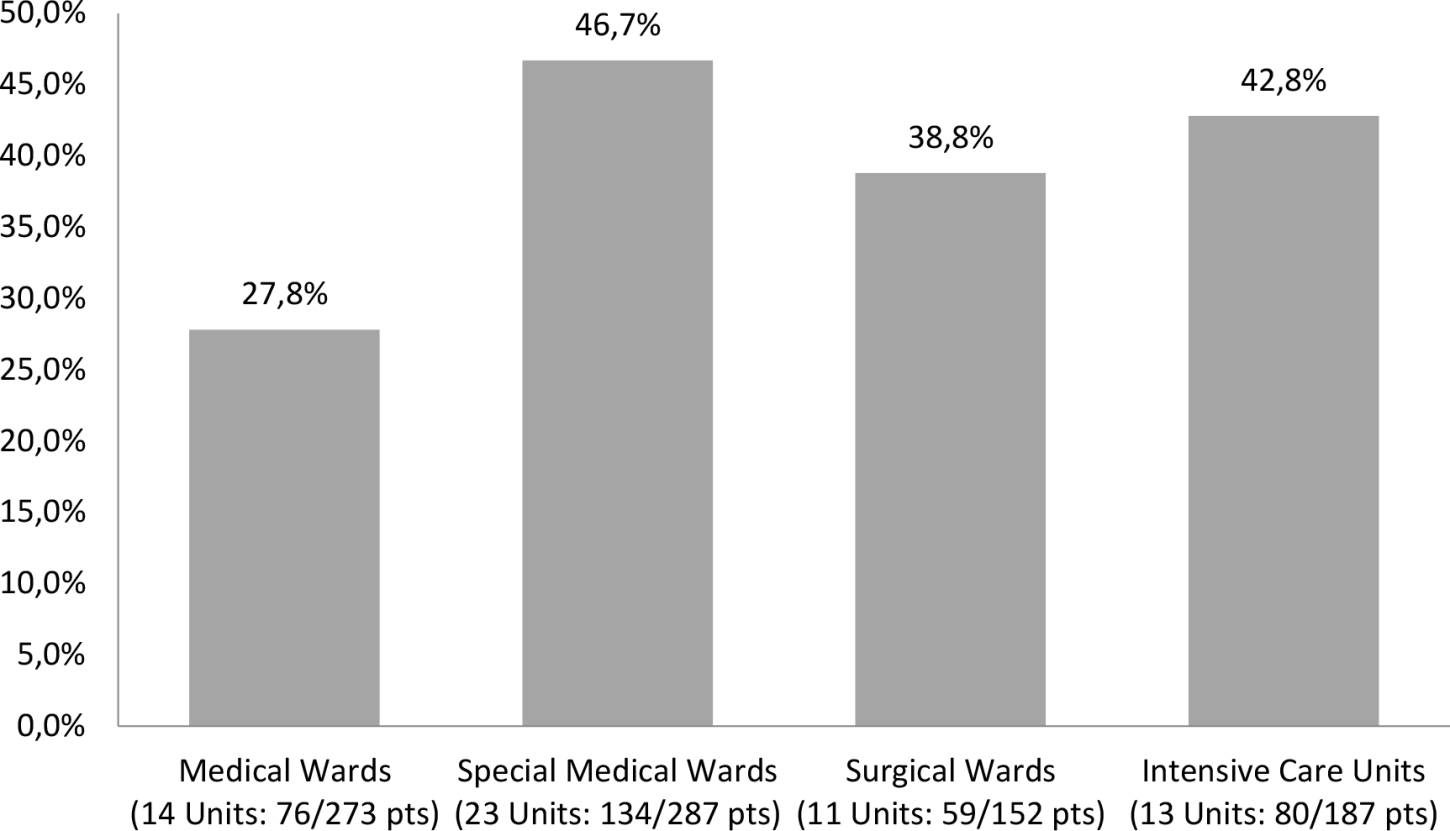
Proportion of paediatric patients treated with at least one antibiotic by ward type.

A wide variability also existed in the proportions of patients treated with at least one antibiotic stratified by ward type. In particular, special medical wards and intensive care units accounted for higher proportions of patients receiving antibiotics compared to surgical and medical wards (“Fig 1”).

Characteristics of all patients enrolled are summarized in Table 2. Median patient age was 24 months and 12.3% were less than 30 days old. Overall, 24.6% of patients were affected by a medical/surgical underlying condition and the most frequent was an oncologic/hematologic disease. Noteworthy is that the rate of oncologic/hematologic patients admitted to the hospital at the time of the survey was 5% (45/899), but this rate increased to 22.3% (78/349) looking at the group of patients receiving antibiotics. These data reflect the fact that oncologic and hematologic patients were responsible for a large proportion of antibiotic consumption in our survey.

**Table 2:** Characteristics of the 349 Italian patients enrolled in the 24-hour ARPEC PPS.

Median age	2 years (IQR 0.5–9)
Neonates	12.3% (n = 43/349)
Children	87.7% (n = 306/349)
Male/Female	201/148
Underlying conditions:	86/349
• Oncologic/hematologic disease	22.3%
• Surgical problem	20.8%
• Chronic lung disease	7.6%
• Respiratory distress syndrome	7.6%
• Chronic neurological condition	7%
• Congenital heart disease	6.7%
• Genetic and metabolic disease	6.4%
• Chronic renal disease	5.2%
• Prematurity and IUGR	4.9%
• Gastrointestinal disease	4.3%
• Other/unknown	4%
• Congenital immunodeficiency	2.4%
• Rheumatological disease	0.6%

IQR = interquartile range

We analyzed antibiotic prescriptions separately in neonates and children (Table 3).

**Table 3:** Demographic characteristics and antibiotic prescription patterns of the neonates and children enrolled in the study.

	NEONATES		CHILDREN	
Department of admission:				
	NICU	36 (83.7%)	Special medical ward	131 (42.8%)
	General neonatal and paediatric department	4 (9.3%)	General paediatric ward	72 (23.5%)
	Special medical wards	3 (7%)	Surgery	59 (19.3%)
			PICU	26 (8.5%)
			NICU	18 (5.9%)
Indications to antibiotic therapy:				
	Prophylaxis for medical problems	24 (55.8%)	LRTI	68 (22.1%)
	Sepsis	13 (30.2%)	Prophylaxis for surgical disease	57 (18.6%)
	Prophylaxis for surgical problems	3 (7%)	Prophylaxis for medical problem	52 (16.9%)
	Skin and soft tissue infections	1 (2.3%)	Febrile neutropenia/fever in oncologic patient	23 (7.5%)
	Pyrexia of unknown origin	1 (2.3%)	Treatment for surgical disease	16 (5.2%)
	LRTI	1 (2.3%)	Other/unknown	15 (4.9%)
			Sepsis	13 (4.2%)
			UTI (upper and lower)	12 (3.9%)
			Upper respiratory tract infection	8 (2.6%)
			Catheter related bloodstream infection	8 (2.6%)
			Skin and soft tissue infection	7 (2.3%)
			Gastrointestinal tract infection	7 (2.3%)
			Pyrexia of unknown origin	6 (1.9%)
			CNS infection	6 (1.9%)
			Joint/bone infection	4 (1.3%)
			Tubercolosis	2 (1%)
			Lymphadenitis	1 (0.3%)
			Acute osteomyelitis	1 (0.3%)
Associations with antifungal agents	8 (18.6%)		48 (15.6%)	
Associations with antiviral agents	0		21 (6.8%)	

### Neonates

**Fig 2:**
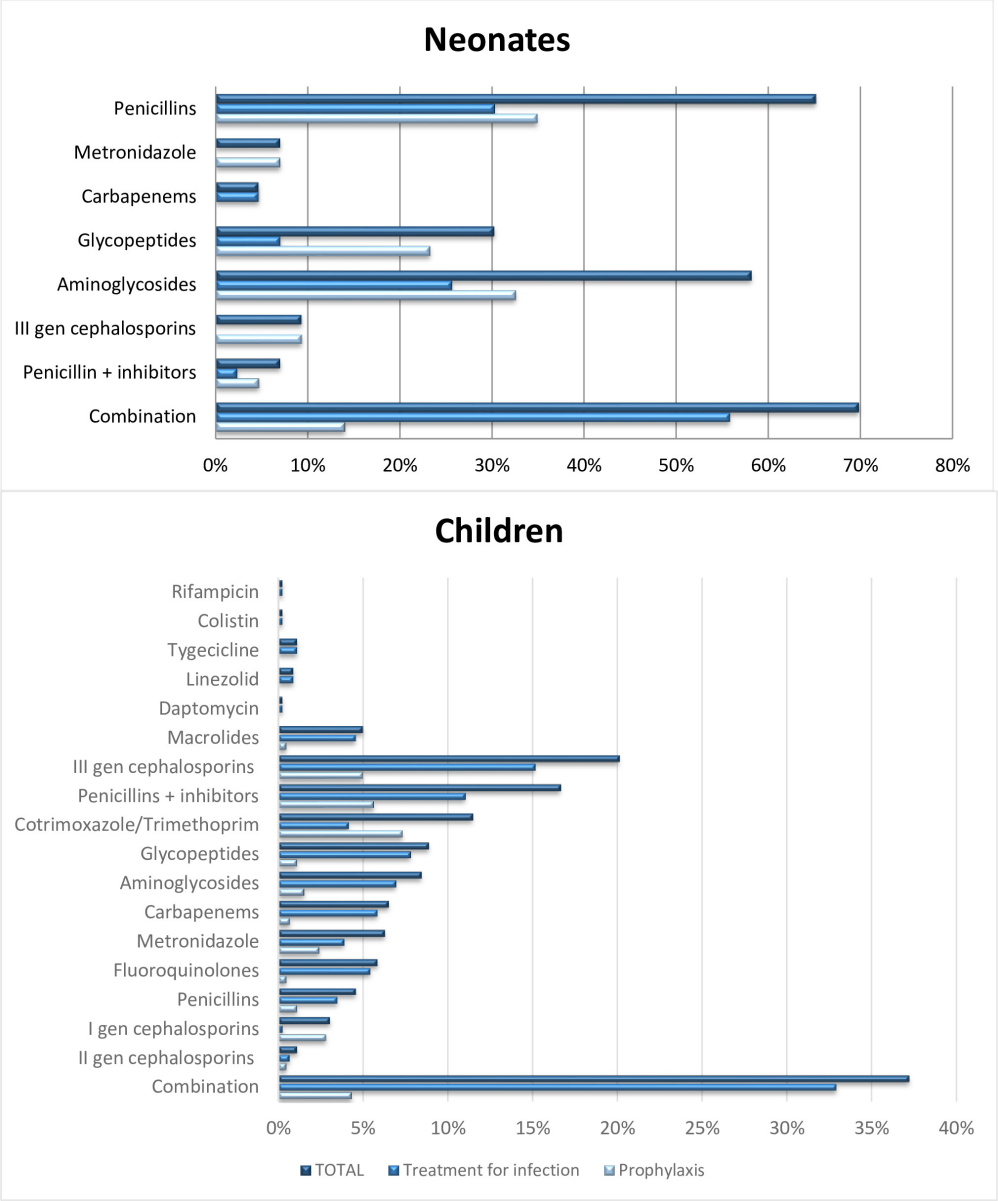
Antibiotic prescriptions among the neonates and children.

At the time of the survey, 248 neonates were admitted in participating hospitals and 43 were receiving antibiotics (17.3%). As shown in Table 3, neonates treated with antibiotics were mostly admitted in the NICUs (83.7%, 36/43). Moreover, 62.8% (27/43) of newborns were receiving antibiotics for prophylaxis and only 37.2% (16/43) were being treated for infection. The top two active antibiotic prescriptions were penicillins (69.8%, 30/43) and aminoglycosides (58.1%, 25/43). Details about indications to receive antibiotics are summarized in Table 3. All antibiotic classes prescribed are listed in “Fig 2”.

### Antibiotic prophylaxis in neonates

The main indication to prescribe antibiotic prophylaxis was medical risk factors (e.g. prematurity, maternal fever during labor, prolonged rupture of the membranes), accounting for 55.8% of all indications for antibiotic therapy in the neonatal subset. The other neonatal patients were receiving prophylaxis for surgical reasons. Monotherapy was prescribed in 10 of the 27 patients on prophylaxis (37%), and penicillin was the most prescribed antibiotic (7/10). Combination therapies in the other 17/27 patients (63%) were variable: penicillins were combined with aminoglycosides in 7/17 cases, while glycopeptides were used with third generation cephalosporins in 3 cases and with aminoglycosides in 3 cases. The last 4 patients received 3 drug combination therapy, including glycopeptides plus aminoglycosides combined with metronidazole (2/4) or penicillin (2/4).

### Antibiotic treatment in neonates

Among all the indications for antibiotic treatment of infection in neonates, the most common was sepsis (30.2%, 13/43). Monotherapy was used just in two cases (one case treated with ampicillin and the other treated with meropenem). Penicillins plus aminoglycosides was by far the preferred combination therapy (8/11), while in the other patients (3/11) glycopeptides were used widely in combination with other classes of antibiotics.

### Children

The paediatric group was composed of 651 patients, 47% (306/651) of whom had an active antibiotic prescription at the time of the PPS. In the group of patients with active antibiotic prescriptions, 64.4% (197/306) were being treated for infections and 35.5% (109/306) for prophylaxis (Table 3). Third generation cephalosporins and penicillin plus enzyme inhibitors were the most commonly used antibiotic classes. More details about indications to therapy are reported in Table 3. All antibiotic classes prescribed are listed in “Fig 2”.

### Antibiotic prophylaxis in children

Approximately half of the children on antibiotics for prophylaxis received antibiotics for surgical reasons (52.3%, 57/109), the others for medical problems (47.7%, 52/109). The most prescribed antibiotics were third generation cephalosporins for surgery (35.9%, 20/57), used as monotherapy in 14/20 cases and combined most often with metronidazole in the other cases (3/20). Cotrimoxazole was the most commonly prescribed agent for the medical problems (67.3%, 35/52), mainly used as monotherapy (30/35).

### Antibiotic treatment in children

The main reason for prescribing antibiotics for infection among children was lower respiratory tract infections (LRTI) (34%, 67/197), followed by febrile neutropenia/fever in oncologic patients (11.7%, 23/197). Focusing on prescriptions for LRTI, 43.3% (29/67) of patients were treated with third generation cephalosporins, followed by macrolides (26.9%, 18/67), quinolones (16.4%, 11/67) and carbapenems (14.9%, 10/67). Cephalosporins were used as monotherapy in 13/29 cases and combined in the other 16 cases, mostly with macrolides (6/16). For 73.1% (49/67) of children with LRTI, the route of antibiotic administration was parenteral.

For oncology patients affected by neutropenic fever/fever, the preferred antibiotics were penicillins with enzyme inhibitors (47.8%, 11/23), followed by carbapenems (34.8%, 8/23), aminoglycosides (26.1%, 6/23) and glycopeptides (26.1%, 6/23). Penicillins with enzyme inhibitors were used in monotherapy in 5/11 cases and combined mostly with aminoglycosides in the other 6 cases. Carbapenem monotherapy was prescribed in 4/8 cases, while combination therapy with anti-Gram positive agents (glycopeptides or oxazolidinones) was preferred in the other cases. The route of antibiotic administration was parenteral in the 95.7% of cases (22/23).

### Use of Carbapenems

Among the 899 patients admitted to the hospital at the time of the survey, 32 (3.6%) were being treated with carbapenems. Focusing on the group of 349 patients with active antibiotic prescriptions at the time of the PPS, 8.9% (32/349) were receiving carbapenems (in particular 4.6% [2/43] in the neonatal group and 9.8% [30/306] in the paediatric group). Considering the overall rates of therapy by department, those with the highest rates of carbapenem prescription were the special medical wards (14.2%, 19/134) and the intensive care units (11.2%, 9/80), compared to 2.6% (2/76) in medical wards and 1.7% (1/59) in surgical wards. Indications for prescription of carbapenems were community-acquired infections in 53.1% of cases, hospital-acquired infections in 37.5% and prophylaxis in 9.3%. Febrile neutropenia was the most common reason for carbapenem prescription (34.8%, 8/23). Therapy was empirically prescribed in 62.5% (20/32) of patients. Carbapenems were mostly prescribed in combination with one or more other antibiotics (65.6%, 21/32), most commonly with glycopeptides (10/21), followed by quinolones (4/21), cotrimoxazole (4/21) and aminoglycosides (3/21). Meropenem was the most prescribed carbapenem, with great heterogeneity in doses and number of administrations recorded. Daily doses of meropenem ranged from 19 mg/kg/day to 129 mg/kg/day, while the mean dose was 70 mg/kg/day. Off-label prescription of carbapenems (i.e. below 3 months of age) was recorded in 18.7% (6/32) of patients and the indications were LRTI, sepsis and surgical prophylaxis.

### Use of Quinolones

In the entire cohort of patients admitted to the hospital, 3% (27/899) were prescribed quinolones. Among the patients with active antibiotic prescriptions, this rate was 7.7% (27/349). None of them were neonates, but 37% (10/27) were below 2 years of age. Considering the overall rates of prescriptions into the departments, the special medical wards (10.4%, 14/134) and intensive care units (7.5%, 6/80) reported the highest rates of quinolone prescription, compared to 6.6% (5/76) in medical wards and 3.4% (2/59) in surgical wards. Indications for prescription of quinolones were community-acquired infections in 44.4% of cases, hospital-acquired infections in 40.7%and prophylaxis in 14.8%. Considering the rates of antibiotic prescription by indication, the most common indication was LRTI (17.6%, 12/67). Among the group affected by LRTI, 41.6% (5/12) had an underlying chronic lung disease including cystic fibrosis and 25% (3/12) congenital immunodeficiency. In general, quinolone therapy was empirically prescribed in 63% of patients. Quinolones were prescribed as monotherapy just in 29.6% (8/27) of patients. In the other cases, they were widely combined with other antibiotics (70.4%, 19/27), especially with third generation cephalosporins (5/19). The mostly prescribed quinolone was ciprofloxacin. Focusing on ciprofloxacin, the prescribed daily dose ranged from 6 mg/kg/day to 30 mg/kg/day, while the mean dose was 18 mg/kg/day.

## Discussion

The 1-day ARPEC PPS provided very useful data on hospital antibiotic prescriptions for paediatric and neonatal patients in Italy. According to data collected in seven large Italian institutions, 38.9% of inpatients received at least one antibiotic prescription during hospitalization. This rate is similar to the mean rate reported from the worldwide ARPEC PPS (36.7%) [18].

To better analyze antibiotic prescription patterns and their appropriateness, we assessed antibiotic prescriptions for prophylaxis and treatment of infection separately.

Our results show that overall 39% of patients were prescribed antibiotics for prophylaxis with the highest rate observed in the neonatal population (63% of neonatal prescriptions were for this indication). The main indication for neonatal prophylaxis was the presence of perinatal conditions (e.g. prematurity, maternal fever during labor, prolonged rupture of the membranes). Prophylactic monotherapy was prescribed just in 37% of neonates and penicillin was the preferred agent, while combination prophylactic therapies including penicillin plus aminoglycosides or glycopeptides plus cephalosporins/aminoglycosides were widely used in neonatal patients. This approach is not in-line with the international literature. Although neonates represent a high risk population due to their immature immune system and the invasive procedure they are likely to undergo in NICU (e.g. indwelling catheters, invasive mechanical ventilation), recent reviews reject the routine use of antibiotic prophylaxis due to lack of efficacy in many conditions [19–22]. Moreover, in 2010 the Center for Disease Control and Prevention revised their guidelines regarding the prevention of perinatal Group B streptococcal disease in healthy neonates, restricting the need for prophylaxis only to well-defined subgroups of patients [23]. Prolonged courses of antibiotics have also been associated with increased risk of necrotizing enterocolitis or death in low birth weight infants [24].

In the paediatric group, the rate of antibiotic prescriptions for prophylaxis was 35.5% of all the prescriptions. Approximately, half of these patients were receiving antibiotics for surgical prophylaxis in accordance with previous European reports in which the proportion of children receiving surgical prophylaxis ranged from 13 to 42% [25, 26]. Third generation cephalosporins ranked first in prescription frequency in this scenario, used often in monotherapy but combined with metronidazole in some cases, confirming their alarming overuse for this indication. This problem in fact was already raised by Ciofi et al in 2008 [16], but a recent paper published by Buccellato et al in 2015 shows that a limitation on the prescriptions of these drugs has not yet been reached [17]. However, it is worth noting that this finding was very variable among the seven centers, since some hospitals preferred the first generation cephalosporins for surgical prophylaxis, as suggested by international guidelines [27].

Cotrimoxazole was the most prescribed antibiotic for medical prophylaxis, used alone in most cases. As explanation, most of the treated children were affected by onco-hematological diseases and cotrimoxazole is the best treatment to prevent *Pneumocystis jirovecii* pneumonia in immunocompromised patients [28].

Regarding the prescription patterns for treatment of infection, the 37.2% of our neonatal cohort was prescribed at least one antibiotic for treating an infection, the main reason was sepsis and the most common antibiotic class was penicillins, combined with aminoglycosides in a large number of patients, in line with international literature [29, 30]. It is hard to compare prescription habits in our centres with other NICUs because of a wide variability of the rate of neonates prescribed antibiotics across hospitals, as shown by a recent multicenter study involving 127 NICUs in the US [31]. The 40-fold variations in prescription frequencies noted in this study did not appear to be related to higher infection burden, necrotizing enterocolitis incidence, surgical volume or mortality rate [31]. They have instead been attributed to frequent inappropriate courses of antibiotics in inpatient neonates, more commonly owing to an unnecessary antibiotic continuation than starting of a non-required therapy [32].

Focusing instead on the paediatric group, we noticed an excessive use of third generation cephalosporins for treatment of infection similar to that seen for surgical prophylactic use, as underlined before. In children with LRTIs, ceftriaxone was the most prescribed antibiotic, used as monotherapy or often combined with macrolides, with a wide total daily dose variability ranging from 12.1 mg/kg/day to 153.8 mg/kg/day. The frequent choice of ceftriaxone as first line therapy for treatment of uncomplicated LRTIs and, in some cases, the high dosage prescribed, are reasons of concern because they are not supported by current guidelines [33]. In fact, other European countries, as the UK and France, seem to have different prescribing patterns for LRTI, preferring amoxicillin/clavulanic acid as first line therapy [34].

An abuse of parenteral cephalosporins in Italian hospitalized children was already denounced in a study conducted by Esposito in 2001 [35] and is a well-known problem also in the adult population [36].

Noteworthy was also the widespread use of carbapenems and quinolones. Indeed, in our study population, among the 349 patients receiving antibiotics, 8.9% were being treated with carbapenems, whereas proportion of carbapenems for therapeutic use reported in the literature in European paediatric units is 4,2% [37]. Though carbapenems were prescribed in most cases for fever in cancer patients, which often requires aggressive antibiotic treatment considering the patients’ immunological status and predisposition to severe infections, we are concerned about the increasingly popular usage of these agents for community acquired-infections, empiric treatment and combination therapy. This is a very alarming finding, considering the doubling of carbapenem resistance rates in invasive isolates of *Klebsiella pneumoniae* reported by the European Antimicrobial Resistance Surveillance Network (EARS-Net)’s report from 2010 to 2013 [9] for Italy. We also found 18.7% off-label use in patients below 3 months of life, but this could be explained by the involvement of many of our centers in the European NEOMERO study, which aimed to evaluate pharmacokinetics, safety and efficacy of meropenem in neonatal sepsis and meningitis [38].

Similar problems were noticed also for quinolone prescription. Quinolones were widely used in our cohort, even if the license for the use of this antibiotic class below 18 years of age is restricted to few rare indications such as cystic fibrosis with pulmonary exacerbations, complicated urinary tract infections, post exposure prophylaxis against inhalational anthrax and severe infections with allergies to other antibiotics [39,40]. Among our patients, the main indication for treatment with quinolones was LRTI. In this group of patients, quinolones were often prescribed empirically and combined with other drugs, though current guidelines do not suggest quinolones as a first-line treatment considering that infections caused by pneumococci or atypical bacteria can still be successfully treated with high doses of β-lactams [41]. Furthermore, the Scottish Antimicrobial Prescribing Group (SAGP) in 2008 and the National Institute for Health and Care Excellence (NICE) in 2015 [42] recommended to avoid the use of quinolones as first line agents for empirical treatment of most commonly infections in primary care, because the overuse of these broad-spectrum antibiotics is associated with a significantly increased risk of *Clostridium difficile* infection [43,44]. The wide use of quinolones in our cohort could be explained by the finding that most of our patients receiving this treatment had underlying chronic pulmonary diseases, such as cystic fibrosis or secondary to immunodeficiencies, but the lack of data about their microbiological status did not allow us to evaluate the appropriateness of these prescriptions.

Our study highlights many feasible targets that need a prompt intervention with appropriate antimicrobial stewardship programs. International guidelines for stewardship identify a wide set of interventions including: disease-specific clinical pathways, audit with feedback and formulary restriction with preauthorization of select agents. The best type of interventions must be tailored according to local practices, resistance trends, and available resources [4]. While the most effective antimicrobial stewardship programs are built on proactive interventions, in settings where a robust antimicrobial stewardship team is hard to establish, clinical pathways tool represent a reasonable and feasible first step for implementation standardizing care without adversely affecting patient safety or outcomes [45, 46]. Moreover, annual PPS could be a useful tool to measure the impact of these interventions on antibiotic prescribing practices [18]. Thus, implementation of clinical pathways in Italian paediatric hospitals associated with annual PPSs could be a good start to reduce the abuse and misuse of antibiotics.

Our study has some limitations. First, data about microbiological isolates and antibiotic susceptibility tests, length of therapies and prophylaxis and previous antibiotic courses could have been useful to better define the appropriateness of antimicrobial prescriptions. Moreover, the characteristics of involved institutions may have affected at least in part the reliability of some results. Most institutions are in fact tertiary care hospitals that usually manage more complicated and severe cases, which might significantly impact antibiotic prescriptions. In addition, the heterogeneity among institutions, in terms of presence/absence of onco-hematology departments or intensive care units, may strongly affect antimicrobial prescription patterns. Finally, the survey was conducted in 2012 and since then a greater awareness of antibiotic stewardship programs has spread among Italian hospitals [47–49].

## Conclusions

Our study took a picture of the Italian situation in terms of antibiotic prescriptions in hospitalized neonates and children and identified many feasible targets that require a prompt intervention to reduce the abuse and misuse of antibiotics. Antibiotic stewardship programs should immediately introduce measures to control prescription patterns in particular for prophylaxis, both in neonatal and paediatric populations, and to limit the over-use of third generation cephalosporins, that seems to persist over-time. Surveillance and educational programs are also needed to restrict the use of carbapenems to more severe conditions. The implementation of disease-specific clinical pathways associated with annual PPSs could be a good way to monitor and ameliorate antibiotic prescription patterns in neonatal and paediatric inpatients over time, in order to reduce as much as possible the worrisome emergence of MDR bacteria in this vulnerable population.

## Supporting Information

S1 File Study database. (XLSX)
